# Extensive retroviral diversity in shark

**DOI:** 10.1186/s12977-015-0158-4

**Published:** 2015-04-28

**Authors:** Guan-Zhu Han

**Affiliations:** Jiangsu Key Laboratory for Microbes and Functional Genomics, Jiangsu Engineering and Technology Research Center for Microbiology, College of Life Sciences, Nanjing Normal University, Nanjing, Jiangsu 210023 China; Department of Ecology and Evolutionary Biology, University of Arizona, Tucson, AZ 85719 USA

**Keywords:** Endogenous retroviruses, Chondrichthyes, Paleovirology

## Abstract

**Background:**

Retroviruses infect a wide range of vertebrates. However, little is known about the diversity of retroviruses in basal vertebrates. Endogenous retrovirus (ERV) provides a valuable resource to study the ecology and evolution of retrovirus.

**Findings:**

I performed a genome-scale screening for ERVs in the elephant shark (*Callorhinchus milii*) and identified three complete or nearly complete ERVs and many short ERV fragments. I designate these retroviral elements “*C. milli* ERVs” (CmiERVs). Phylogenetic analysis shows that the CmiERVs form three distinct lineages. The genome invasions by these retroviruses are estimated to take place more than 50 million years ago.

**Conclusions:**

My results reveal the extensive retroviral diversity in the elephant shark. Diverse retroviruses appear to have been associated with cartilaginous fishes for millions of years. These findings have important implications in understanding the diversity and evolution of retroviruses.

**Electronic supplementary material:**

The online version of this article (doi:10.1186/s12977-015-0158-4) contains supplementary material, which is available to authorized users.

## Findings

Retroviruses infect a wide range of vertebrates and cause many notorious diseases, such as AIDS and cancers. However, much remains unknown about the diversity of retroviruses in basal vertebrate species. In particular, only several retroviruses have been identified in fishes, including Snakehead retrovirus, walleye dermal sarcoma virus, walleye epidermal hyperplasia virus, and Atlantic salmon swim bladder sarcoma virus [1-4]. Retrovirus employs a unique replication strategy, which requires reverse transcription of its RNA genome into DNA and integration of viral DNA into the host chromosomes. Occasionally, retroviruses infect germ line cells, and the resulting integrated retrovirus, known as endogenous retrovirus (ERV), becomes vertically inherited as a host genomic locus. Over time, some retroviral insertions are fixed in the host population. ERVs provide important insights into the ecology and evolutionary history of retroviruses.

Cartilaginous fishes (Chondrichthyes) are the most basal class of vertebrates from which retrovirus has been reported [5]. Here, I analyzed the recently available genome sequence of the elephant shark (*Callorhinchus milii*), a high-quality genome assembly covering approximately 94% of the *C. milii* genome, for retroviral insertions [6]. The tBLASTn algorithm with various representative retroviral Pol protein sequences was employed to screen the elephant shark genome for candidate ERV sequences. To distinguish ERVs from other LTR-retrotransposons, I used a strict criterion: only the retroviral Pol protein homolog sequence with a downstream Env protein homolog is defined as an ERV element. After initial identification of ERVs, the BLASTn algorithm was used to identify short ERV fragments. My genome-scale screening procedure identified three complete or nearly complete ERV insertions (within the *C. milii* genome scaffolds 2, 324, and 2324, respectively; Additional file 1: Dataset 1) and many short ERV fragments in the elephant shark genome. I designate these retroviral elements “*C. milli* ERVs” (CmiERVs).

**Figure 1:**
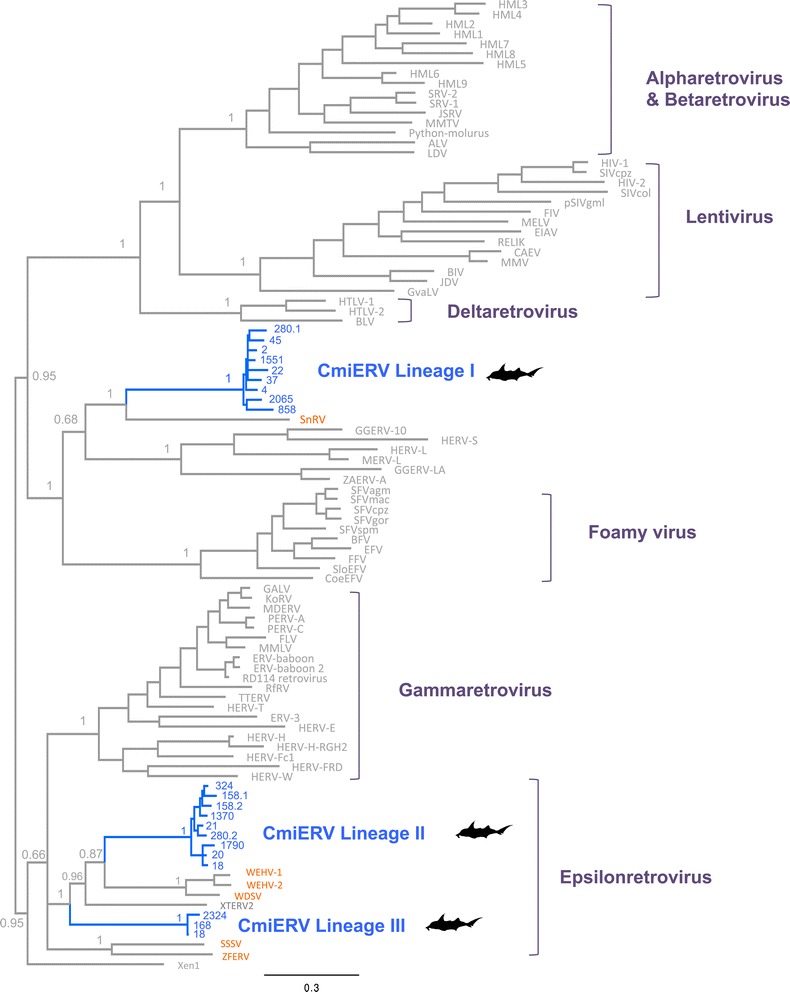
Phylogeny of CmiERVs and other representative retroviruses. The phylogeny was reconstructed based on the retrovirus Pol protein sequences. Posterior probabilities are labeled near the selected nodes. The CmiERVs and the retroviruses of fish origin are highlighted in blue and orange, respectively. The numbers of the scaffolds where CmiERV fragments were identified are labeled near the corresponding tips. Abbreviation: ALV, Avian leukosis virus; BFV, Bovine foamy virus; BIV, Bovine immunodeficiency virus; BLV, Bovine leukemia virus; CAEV, Caprine arthritis-encephalitis virus; CoEFV, Coelacanth endogenous foamy virus; EFV, Equine foamy virus; EIAV, Equine infectious anemia virus; ERV-baboon, Baboon endogenous virus; FFV, Feline foamy virus; FIV, Feline immunodeficiency virus; FLV, Feline leukemia virus; GALV, Gibbon ape leukemia virus; GGERV, *Gallus gallus* endogenous retrovirus; GvaELV, *Galeopterus variegatus* endogenous lentivirus; HIV-1, Human immunodeficiency virus type 1; HIV-2, Human immunodeficiency virus type 2; HML1-9, Human MMTV-like 1–9; HTLV, Human T-cell leukemia virus; HERV, Human endogenous retrovirus; JDV, Jembrana disease virus; JSRV, Jaagsiekte sheep retrovirus; KoRV, Koala retrovirus; LDV, Lymphoproliferative disease virus; MDERV, *Mus dunni* endogenous retrovirus; MELV, *Mustelidae* endogenous Lentivirus; MERV-L, Murine endogenous retrovirus type L; MMLV, Moloney murine leukemia virus; MMTV, Mouse mammary tumor virus; MVV, Maedi-visna virus; PERV-A, Porcine endogenous retrovirus A; PERV-C, Porcine endogenous retrovirus C; pSIVgml, Gray mouse lemur prosimian immunodeficiency virus; Python-molurus, Python molurus endogenous retrovirus; RELIK, Rabbit endogenous lentivirus type K; RfRV, *Rhinolophus ferrumequinum* retrovirus; SFV, Simian foamy virus; SIV, Simian immunodeficiency virus; SloEFV, Sloth endogenous foamy virus; SnRV, Snakehead retrovirus; SRV-1, Simian retrovirus 1; SRV-2, Simian retrovirus 2; SSSV, Atlantic salmon swim bladder sarcoma virus; TTERV, *Tursiops truncatus* endogenous retrovirus; WDSV, Walleye dermal sarcoma virus; WEHV-1, Walleye epidermal hyperplasia virus type 1; WEHV-2, Walleye epidermal hyperplasia virus type 2; XTERV2, *Xenopus tropicalis* endogenous retrovirus 2; ZAERV-A, *Zonotrichia albicollis* endogenous retrovirus type A; ZFERV, Zebrafish endogenous retrovirus.

To assess the relationship between CmiERVs and other retroviruses, CmiERV and representative retroviral Pol protein sequences (Additional file 2: Table S1) were aligned using MUSCLE [7]. The ambiguous regions in the Pol protein alignments were removed using Gblocks 0.91b and then manually edited [8]. Phylogenetic analyses were performed using MrBayes 3.1.2 [9]. My phylogenetic analysis shows that these CmiERVs form three distinct lineages (Figure 1). Lineage I CmiERVs cluster with the retroviruses isolated from the snakehead fish (*Ophicephalus striatus*), while lineage II and III CmiERVs cluster with the epsilonretroviruses isolated from the walleye (*Sander vitreus*) and amphibians. CmiERV lineage I and lineages II/III are only distantly related to each other. These CmiERV lineages are likely to result from three independent retroviral invasion events. My analysis provides clear evidence there is extensive retroviral diversity in the elephant shark. It is possible that exogenous retroviruses related to CmiERVs identified here are still circulating in the elephant shark and possibly other Chondichthyes.

On endogenization, the 5′LTR and 3′LTR of a nascent ERV are identical and will accumulate mutations independently. Thus, the 5′LTR and 3′LTR genetic divergence could be used to estimate ERV invasion time [10]. The invasion time of an ERV can be estimated through:

$$ t=\frac{d}{2u} $$

where *t* indicates the invasion time, *u* indicates the neutral evolutionary rate of host, and *d* indicates the genetic divergence between 5′ LTR and 3′ LTR. In this study, two complete CmiERV insertions were identified (Table 1). The genetic divergence between 5′-LTR and 3′-LTR was calculated with the Kimura two-parameter substitution model [11]. The neutral evolutionary rate for the elephant shark is not available but is approximately an order of magnitude lower than those for mammals [12]. The average neutral rate for mammals is estimated to be 2.2 x 10^−9^ substitutions per site per year [13]. I thus assume 2.2 x 10^−10^ substitutions per site per year as the shark neutral rate. Then the insertion times for the two complete ERVs are approximately 75 and 54.5 million years ago, respectively. However, these estimates should be taken with cautions [14], given that I am not sure whether LTRs evolve at a neutral manner and what the actual neutral rate for the elephant shark is. Nevertheless, these results suggest these retroviruses were infecting the elephant shark millions of years ago.

**Table 1:** **Genomic position and invasion time of two complete CmiERV insertions**

**Complete CmiERV**	**Lineage**	**Genomic position**	**5′- and 3′LTR divergence**	**Time (MYA)**
1	I	Scaffold 2: 5,443,211-5,452,770	0.033	75
2	II	Scaffold 324: 74,676-84,761	0.024	54.5

Previously, a single ERV sequence was identified in the lemon shark (*Negaprion brevirostris*), which is closely related to human ERV; this ERV was thought to have a cross-transmission origin [5]. However, I find that CmiERVs cluster together with retroviruses of fish origin. The phylogenetic pattern is compatible with the hypothesis of an ancient marine origin of retroviruses [15]. Chondrichthyes are the most basal class of vertebrates from which retrovirus has been identified; no retrovirus is identified in earlier-diverging vertebrate lineages, the lampreys (Cephalaspidomorphi) and the hagfish (Myxini) [5]. It follows that these CmiERV elements are likely to represent “primitive” retroviruses. However, the possibility that these elephant shark retroviruses originated from cross-transmission from other fishes cannot be formally excluded.

To date, only a limited number of exogenous/endogenous retroviruses have been identified in fishes [1-5,14]. My results reveal the unexpectedly extensive retroviral diversity of the elephant shark. The initial candidate ERVs were identified based on a strict criterion – whether there is a downstream Env protein homolog following the Pol protein homolog. This approach is conservative, given that the retroviral Env protein evolves rapidly and its similarity to other retroviral Env proteins will erode over a long time. On the other hand, the ERVs identified using this approach are authentic retroviruses. It is likely that there are additional ERV insertions that were not detected. Also, it should be noted that only a small proportion of retroviruses could leave endogenous copies in their host genomes [16]. Therefore, I believe the actual diversity of retroviruses is more extensive in the elephant shark. Further analysis of ERV in basal vertebrates would improve our understanding of the diversity and evolution of retroviruses.

## Additional files

Additional file 1:
**Dataset 1.** Sequences of three complete or nearly complete CmiERVs. Additional file 2: Table S1. The representative retrovirus sequences used for phylogenetic reconstruction.
