# Supplementary material for: Extensive retroviral diversity in shark
Source: Retrovirology. 2015 Apr 28;12:34. doi: 10.1186/s12977-015-0158-4 (PMC4422223; doi:10.1186/s12977-015-0158-4)
Supplement: Additional file 1: — Dataset 1. Sequences of three complete or nearly complete CmiERVs. [file 12977_2015_158_MOESM1_ESM.pdf]

Dataset 1. Sequences of three complete or nearly complete CmiERVs

>CmiERV_Scaffold 2
TGTTAGAATTTACACTTTTTGGATAATGTTAGAATCATAATAGATGTTGCGAACGGGTT
AACGTTTAAGTTAAGTGTGGGGGACAACTGGACTGACAGTCATGGGATAGGACGCATCT
GTTTTTCTCTGTTTTTGAAAGAATAACGGACAGGGGCAGGGGGTCACCTCTTATCAGTA
AATGACAACAAGCCACAGAGGTAACGGTCCAAGGTACCGGTCGACATGGGATCTTGACT
GAGCAATAAGTCGCTAGATTTCTGACAGGGCCTGCCAACCGACAGAAGGCAACCCTCAAG
AATAGCAACTAATGAGCAAGTTGTTTAAACAAGAGGACCTTGGTACCATCCCGGGATAC
TGATAAGGAATAACCAGTCACTTAAAAGGAATGTACTAATGATCCCACACCACAAAATA
ACAAGCATCAGATACAGCTCTAAACTGATCAATCCTCTATCTCAGACAATTAAAGACAAT
GTGTATGTGGGCTGAGGCACGTACACCCCACGAGGTGTTCACGTGAACCCAGCATGATTT
TGGCAAGGTGACCTAATGTCCGCACCATGATTAAGACACAGTGCAAGACTAAGATCGAC
GCGCGCCCAGGACAGTACCTACCGTGGATGGAAGACTGAGAGCTAGAAGACAGAAGTAG
ACGACCACGAGGAGGAATTACGCGGTAGTATTGTTCTGTTAGTAAATTGATTGATTGTG
CTTACTTGCCCACGCCCATCGGGAACTTGGACGGATCCTGGAGTTTAATAAACACTACTC
TGGTTGCAAACAGTGGTTGTGTTTGATTGGAACTCGAAAAGAAGATTTTATTTTCTAAC
AATTTGGCGTAGTCGGCAGGATCATTCAGGAAAGTGGGACGGAGTAAGTACAATCAAGC
CTTGACGAAGGTTTATGTTAAGTTTTTAGTTAGTTACTTATAAATGTTTAGTTTTTAGT
TATTCCCCTATAGGTGTTGTTAGTCCAAACTGAGGGAAGGAAATCCCTTATTACCTCCGG
GTAAGAAGAGGGACCAGGACTGATAGTAATTGAATGTTGATTTAAATTGTGACTGTGGT
CGCTGGAAATAACCTGTTGGTTGAGACTGAGCTGTGTTAAGTTAATCCAAACTGAGGGA
AGGAAATCCCTTATTACCTCCGGGTAAGGAGAGGGACCAGGACTGATAGTAATTGAATG
CTGATTTCAATTGTGACTGTGGTCGCTGGAAATAACCCGTTGGTTTTGACTGAGCTGTGA
AATTCGAAAGAAAGCTCATTAAGAGAGTTAGTACCGTGAGTCGTGGGGTAAATTTCAAC
TGTTCGGACAGCCAGTGCAAGCTGAGGAAAACCCTCTATTACCTTGGGCAAGGAGAGGG
ACCAGGACCGATAATAATTGGCACTATGGCCCCAGGAAATAATTCTGGTTGTGACTGGGC
TGTGAACTTTGAAAGGAAGCTCATTAAAACAGTTCAGAAGTTGAAGTTGGGGGCAAATG
ATGAGAATATTCCGCAGCCAAGTGGCGCTACAGGGTATAAGTCTTTAGTAGGAGAAATC
GAGGAGTTAGGCACTCAGCGGAGGTTGAGTGATAACAAGAAGAAGGTTTTGTTGAAATA
CTGGGCTGTCGTGAGTTGCCAACACCTAGAAGAAGAAAGACACATATGGCAGGAGGAGA
AAAAATGTGAACAGGAGAGATTAGAGGAGGAGCTAGGAAAAGTGAGAGAACAGGCTGCT
AAGCATGAAGCAGATCTTCATGCCCAGCAGGAGCATGAGAGGGAAAGATTTCAGAAGGA
TCTGGAAGAAGTGAGAGAACAGGCTGCTAAGCATGAGGCAGATCTTCATGCCCAGCAGG
AGCATGAGAGGGAAAGATTTCAGAAGGATCTGGAAGAAGCGAGAGAGCAGGCAGCAAAG
TACAAGGCGGACTTCTACGCTCAGCAAGAGCGCGGGGAGTTAGAGCGAAGGGACTTAGA
AAGTGCGAATAAAGACATAATTGAGAGATATACTACCCTGCAGGGAATTTATAACCATC
AAGGCGCTATTTTAAATCTAACGGTTGTTAAATCTCGAGAGGTCGAGGAGCTTAAGGAA
GAACGGTTGCGAGATAAGGAAATGATTGCCGGGTTGAGACAGGAAAATGATGATTTGAG
GGGAGCCCTGCGCTACCAAGAAATAGGTAGGCAGGAGCGAGCGGAGTTAGAAGAGTTAA
GGAAGAGAAAAGAGGGAGAGAAGGAAGGTAACGAACGGAGACAAGCGTTAGTAGCGGCG
GTATGTGACATTAAGCCAGTGATAAATGGGGTTTGTCAGGATGCCGATGATAATAATGT
AGATTGGGGAGAGTTGGATGAAGAGGCAGTGCAGTACAATGAGGATAAAGGGTCAGGGC
CACCCCTAGTAAAGGGTCGTGTTTGTCCGGTCAGCACTTCACAGCGTACCAATGTGAATG
CTGGGGGCCGGGACAAGATTATCAACTCCCTGAGCCCGGGAGATGTGGTAGCGTATTGTT
CTCAGCTGGGGCCCAAATTAACTCAAGGAACCCCATGGCAACCCTGGATAATGAAATGGG

ATACTTGGAAAGAGGATAACGGTGACTTAACTCCCGCCGACTATGCTAGGACACTTAAG
GCAATGTTAAGCTCGGATCTCCGAGGGGCTTTAGACCCTCGTACGATCCCAGGCGGAATT
GTTACCCCTGCCAATTTACAGGATTTCGAGGCAGAGGTGAGGCGAGAGCTGGCTGCCCAC
TCCAACTCCCTTGAAGATTTGACCTTGTGTAAGCAGGGGGCAAATGAAAGTCCCCTGGCT
TTTCATCTCAGACTGTGGCAGCTATATTCGGCTAATCAGGAAAATGCCATGAATCGGCAA
CAATGCGAGAATTCTGTTAATTATAAAAAATGCTGGTTAGGGAAATTGAATAAGAATAC
CTCTGCTGCTATGGGGTTAGGCATAGATCCCGCCTCCCTTTTTCATCAACTGATGGGAAC
AGCCACTAGGTGTCACGGGGCTATTCAGAAAACTATTAAGAGTGAAAAGACAGAATCTG
TGTGGCAGGTGGAGGGGCGAGAGCTGTGGAGACCAGGGAATAATGGTCCGAGCCGAGGG
CAGCCCCGAAATCAGGGACCCCGAGATAGTTCAAGGGATACATGCTATACCTGTGGTAAA
CAGGGCCATTGGGCTCCGGACTGTCCAACAGGGCGCAGAGCCCGCGCACAGAGAGGGCAG
AGCTTTTCTGCTGACCCCAAACCTGATCAAGAACAGCAAAACTATGAGCAATGGGAGAG
GAACCTGGAGCAGAAGTTAACCCAAGGAATCTTGACTCAGCTAGGCCATAAGCCAGGAC
GTGGAGGTGGCTCTGTCTCGGCTATAGTCGAGGGGAACCGACCATGCCCTCAATGACATG
TTTCGGTCCCATACCCTGATGGCAGCCAAGTGAGCAATTGGTTGTGTGTCCCCTTTGAGA
AATACGAGGGACCGAGGCCGTTTATATCCATGAAGGTGTGCGGGACCGTCCATGCTATTT
TGGTAGATTCAGGCTCTACAGCCACCCTGCTTAATTTCGAAAACCAACCCCTTCCTCCCC
TCACAGGTGAATGCTTGGAATTTTCTGGTGTGTCTGGTAGCCCTATGTGTTGCGTCCTTT
GCCTGTACAATTGACTTGTAAAGCAACGATTGTTTTAAAACAGCCAATTGCTGTAAATT
TAACCACTGAACCTGGTATCCTCGGGAGCGATGGAACTAACCTCGCTTAATGCTCTGCTT
GACTATACTAATCATTGTTTATGGTTAGATCAACATCCTGGTCAGGATCGGTTTTGTGA
GATATTAGATCGGCATGCTGTGTGCGCTATTATAAAGCCCACCCCATACCCTCCAGTTCA
GTCAAGTCACCCCTGGATTGGTCCATTATTATCCCAGTTTCCAGGTCTCCGAGCCACTCA
CAAACACGACTGTGGGAGGAAGATGGCAGTACCTGAAGAGGTTAATGGTCCCACCCACCA
GCCCTGTAAACAATACCCTTTGCCTAAAGAGAGTTTACCTTATGTCCAGGCCACAATTGA
GAGTTTAAAGGAACAAGGTGTGATTTGTGAAGGGATATTTACTACTAACTCCCCTATAT
GGCCCGTTAGGAAACCTGATGGTTCCTGGTGGTTAACCATTGATTATCGGCAGCTTAATA
AAATTACACCTGCTTGTGCCCCAGTTGTAAGTGACATGAGCCAGCTTCTGAAGGACATCC
CGTCTACTGCTAAATGGTTCTCGATCCTGGATATTTCAAACGGATTCTGGAGCATTCCTC
TAAAACCAGAGGACCAGCATAAGTTTGCGTTTACGTTCAATGGTCAACAGTACACTTGG
ACAGTTCTACCTCAAGGTTTCCATAACTCTCCGACCATATTTCACAAACGTATGGTTCAG
GCACTGCGTAATGTTGTTCCCCCTGATGGTATGAGTATTCTCCAGTTTGTTGACGATATT
TTGCTAATTTCTGATACTCAGGAAACTCATCAGACTCTCCTAGAACGGGTATTGAAAGCG
CTTCTTCAGGCTGGATTGAAACTTAACCCCATGAAGGTACGGTTCCAGCTGTCTCTTTTC
TAGGTGTACAACTGGATGCAGAGGGACTTCGACCCGATGCTCATAAGGTCGACATCATTC
AGCGCCTCCCACCTGCCTGTGACCAAGACTGCACTTCGTTCCTTCCTTGGACTGGTGGGG
TACGAAAGGTAATTTCTCCCACAATTTGCTGAACTGGCCAAGCCGCTGTACAACCTTGTT
AAAGGCGATATCCTTGATGATTTCCGTGAACACTGGACAGATGAACATACGGAAGCCGT
ATCTCAACTTAAACAAGCAATGATGCAGGCTGCTGCTTTGGTGCACCCCCAGCCTGATCA
GCCTTTTCACTTAGAGGTTGCCGGAGGTCCTACTGCATTAGCAGCCGTTTTGACTCAGCA
GTTTGAAGGTCGGTACCTCCCGATTGCATACATATCTCGCATTCCCACTGGCGTGGAGCA
ACAGTTTGATCCGTGTGAACGTCACCTTTTAGCCGTGTGTTGGGCAGTCACCTCCTTTCA
ATACATCACTGGACTCCAGAAGATTATTCTTCATTCTCCACACATCCCCGTTCGTCTCCT
GCAACGACCGGGCAATATGCTTGTCTCCTCACAAAGACTCACTAAATGGGCTCTCACTCT
GGCTGCTGCTGATTTACCGGTTGTTACTGGTCTGACTACCCTCATTCCCCAGATGATGTT
GTAAGACGGTGACCCGCACACATGCCCTCTTCCGTGCTTGAACTTCAGTGATACTGGGAT

CCTTAAGTCCCCGCTGGAGGCTTCCCCGGTGCAGTCTGTCGCCAATGTGTACATTGATGG
CTCCTCCTTCCATGTTGATGGCAAGGCATTCACTGTTTTTTCCGTTATCCTCCCTGATTG
TGACATTAGTTTCCGCTGCATACCACATTCTGCTCAATATGCTGAGTTGGCCGCACTCGT
TGAAGCGGTACGCCAGACTGCCAAAGTGGCTACCAACATATGGTCCGACAGCGCTTATGT
TGTAAATACTGTGTTACTATTACCTTTGTACTCTTCCCGAAATTTTTGTACCTTTGCAGG
AACGACGTTGGTTCACGCGTCACTTCTGAGATGTCTGGCTGATGAGATATCCTGTCGCAC
CCATCCGCTGTATGTTGGTAAGGTGGCAGGTCATCAAAAGACTGGACCGCACTCACACAG
GAACAATCATGCTGATGTCGTTGCTCACCATTCCGCTCTTAATGGAGAAGCATGGATTTC
TTCCCCTCTTCCGTCTGCCTCAGTGAGGAGCATCTCAACTTCGCCCTCTTTGACTGATTTG
ATTAAACTCCAGAATTCATACCCGCCTTACATCGAAGCCGTGCAGTGGCACCAGGAGCAA
CTTGCTGGACTTGATATCCATCCCGATCCACAATATGATTGGCTAGTGCACACCCATTTG
TACGTTCCTCTGGACTCTCTGGCCTCCCCTGTTCTCATTCTCATGTATTCTAAGCACGAT
ATCTCCACTGTGTGTATTCCCACAGATTGTGCTGATGCGATCTTTCGGTTATACCACTCA
CATCCTACTGGTGGTCACTTTCGTCCCTCAAAAACCATCACTAAGTTGTTGCGCTCCGTC
TGGTGGCCTGGTTTGTCTCATGACGTTCATGAACGATGTAAGACGTGCCTTGTGTGCTTC
CAGCATAATCCCCCTCGATCTACTAATCGAGCGTTATTGCAGAGTGCACCCTATCCCTCA
GGTCCTTGGACCAATATTCAGATTGATTTTATTGGGCCATCGTTCACAGCTGCCGGTGGT
TACAAATACTGTTTAGTCATTATTGATAAGTTTACAAAATGGATTGAAGCTTTCCTGAC
TAAAAATAACACAGCGCTCACTGCTGCACGCATTTTATTGCGTGAGGTGATCTGCAGGTG
GGGAATCCCTGGTATATTGGATAGTGATCAGGGATCACATTTCACGGGCAAAGTTTTTA
CTAAATTGATGGAATTGCTGGAAATTGATCACAAATTGCATGTGTCATACCATCCTCAA
TCCTCAGGGGTAGTAGAAAGAAGTAACCGCACATTAAAGGTTTGGTTGTCAAAATTTTG
TGCAGATCACCCCACGCAATGGATGAAGGGATTGCCACTGTGTCTAATGGCAATTCGCTC
CATCCCACATGCTACTACTGGCATTTCACCCTATCAAGCGATGACAGGTCATTTAATGAG
GCTACCAGGTTGTACAGTAGTGATGACTGAACAAGCCCTCGAACAATTAGAGGGTAATG
TTAAATTGCAGGGAACTGCGGAGGAGACATGGGTGAAAGAGATGCTAAAGATCATACAG
GAAACCAGCAGGTTTGTGGCTTCAAACACTGGTAAGGCGAAACAAAGTATGAAGCAACA
ATTTGACACTACCGTTAACCATGTGGAGTATGAAATTGGAGATGAAGTCATGGTCCGCG
TGTATGGGAAAAAGGAATCTCCTTTTGCCCCGCGATGGAAAGGGCCTTTTGAAATTTTG
GAAAAGGCAAGCCCGGCAGTATATTTAATTGCCACGCCTTTGTCTTCTAAATTGACTAAA
TGGTACCATGTGAATCAACTCAAAACTGTAAAGAAGGGAAAAGGATAAACTGATGACTG
AATATATGCTACATTTGTATTTTGCAGGCACCAACCGAGTGATTACCGGATCTTCTGTAT
CAATATGGATCTGGATGGAATTACTGGAGGACTGGTGCTTGGCTTTGTAATACTACTAA
TCCAGAACTCCCACGTTTACTCCCATGATAACCCCTTTGATGGAAACCCCCATAATACTT
TCTTATTAGCAGCTTATAGATATGCAAAAAATAACCAGGTGTCTAGTTGTTATGTATGC
GCTAATATGCCAACATCTGTTAAACATGGAATACCAGTAAGGGCCATTCCTGGCAATGTT
AGCTTAACTGCATTTTGGTATTTCAACCAAAATCAGACGAATGTTAACCACGCCGCTGCA
TGCGACTGGTCAATTCCTATTGGGGACGATCGAAGACGATGGGAATCAGCAGGGTATAG
TTTCAAGGAATACCGTGGGTATTACCAACCACCATATGATGTTACCCTCTCACCCCCGAC
ATTAAGTGTTCCCAACTCCCCTTCAGGTGAGATCTGCATCTCCCGCAATATAACTGGGGG
ATTTCCTGTAGGAGAGAGCAAATGTGCCCAGACATTTGTGATAGGTAACGATACGTGTT
CCCACCCACGGCATTGGCCATATACCATCCTCAGTTTCAAAAATCTCACCACAGGTACTT
CCTTTAACCAAGTTTGGATTCCCCTGAGGTTGGGACCCAACATTACTGCTTATAACAACA
CTTATTTTGTATGCGGAAACAAGGCCTATCCATGGCTGCCGCTTAATTGGACAGGGAAAT
GCTATCTTGCTTATGTTGTCCCAGGTCTAAGACATGCCTATAAGTTACTAAATGACCACC
ATATTAACAAAAGATCCATCACTAAGGCCGAACCATTCTTTGCTATTGCTTTTCCCATGT

ATAGAACTTCTAAGTTTTCCCAAGAAAGCATTAGTATGGCTAAAATCCTTGAGCAGGTG
GCAAATGATACCGCAGAAGCTGTTGACTCTTTGACCGCTGAAGTAGTTGCTATTCGGACA
GTGGCTTTGCAAAATCGCATGGCATTAGATTTCGTATTGGCTAAAGAAGGAGGTGCATG
CGCCATAATTGGGTCTGAGTGTTGTACATATATTCCGGATAACTCCGAGAATATAAGTA
GATTGACTGATCATATCCGGAAAGAGATGGATAAATTGAAGCAGGCCCCATCTTCATCTC
TTTGGGCATGGACCACTTCTTGGTTGGGTTCTATTGGAAGTTCACTGTTACATGGGCTAA
TTATTATTGTTGTGATCTTGCTTTGCGTTTATTGCGCTGTTACGGGTGTGAAGTTTATTT
TTCTTAAAGTTATGTCTAGTGTGGTTTCTTCTGAGGTACCCTTTGTAGGGAATGATGGA
GAGGCTGGCAAAGTTGCTGCCATCCACCCTGTTGACTGGAAGTACCATCCTCCCTCCCAG
GTTGTGTGACCTTGTACTATTATTCTGACTTTGTATTATTTGTCTTTTTGTATTATCAGG
TTGTGTAACCTTATTATTTTCCTGATTTTATATTATATTCTTTTTGTAGTTTTGTGATAC
CTCATGTTCATTCGGTATCAAGGTGAGGACTGTTAGAATTTACACTTTTTGGATAATGT
TAGAATCATAATAGATGTTGCGAACAGGTTAACTTTTAAGTTAAGTGTGGGGGGCAACT
GGACTGACAGTCATGGGATAGGACTCATCTGTTTTTCTCTGTTTTTGAAAGAATAACGG
ACAGGGTCAGGGGGTCACCTCTTATCAGTAAATGACAACAAGCCACAGAGGTAACGGTCC
AAGGTACCGGTCGACACGGGATCTTGACTGAGCAATAAGTCGCTAGATTTCTGACAGGGC
CGGCCAAGCGACAGAAGGCAACCCTCAAGAATAGCAACTAATGAGCAAGTTGTTTAAAC
AAGAGGACCTTGGTACCATCCCGTGATACTGATAAAGAATAACCAGTCACTTAAAAGGA
ATGTACTAATGATCCCACACCACAAAATAATGAGCACCAGATACAGCCCTAAACTGATCA
ATCCTCTATCTTAGACAATTAAAGACAATGTGTATGTGGGCTGAGGCACGTACACCCCAT
GAGGTATTCACGTGAACCCAGCATGATTTTGGCCAGGTGACCTAATGTCCGCATCATGAT
TAAGACACAGTGCCAGACCAAGATCGACGCGCGCCCAGGACAGCACCTACCGTGGATGGA
AGACTGAGAGCTAGAAGACAGAAGTAGATGACCACAAGGAGGAATTACGCGGTAGTATT
GTTCTGTTAGTAAATTGATTGATTGTGCTTACTTGCCCACGCCCATCGGGAACTTGGACG
GATCCTGGAGTTTAATAAACACTACTCTGGTTGCAAACAGTGGTTGTGTTTGATTGGAA
CTCGAAAAGGAGATTTTATCTTCTAACA

>CmiERV_Scaffold 324
TGTGGAAGGTGAATTCAGCACATTCTGCAGAGTTAGCAAAATGAACAGGACGTGAGGAC
CTAAGACCAAAAGGGCAAAAGGCCAGGGGGGCCACTGGCCTTAAGTGAAATATATGCTG
TCATGAAGTTGTCACGAATGCTCGTGATTGGCATTGTTTAAAGATAGATAAGCAGGTGT
CTAGTATCGGTACCGCATCCTGAAGTCATGTCTAGAATCAGTTCTGGGGTCAGATAACAT
TCAGCAGCATCAGCATTTGTCATTCTTTAAAATGTCCTTTTCCTATATGTACTTAATTGT
AAGGACAGAGTGATTGTCTTTGCAAAAACAGACATCCGGTCACAAGGAAGACTATATCG
AAATGTAACAAATTTAGAGGGTCCCCTCATGATTATTGATTGACTCATAGTTCCATAAG
ATTTTGTATAAAGGTGAGGTGCTTGCTCCCATTTAGTGTGTAGTGTTGGGAGCAGCACA
CGAGCAGTTGTTGCCACGATACTACACCCCACATGTGTGAAAATAAAGCTTGTTAATTGA
ACGCCGCACTGACTCCGTTGTTGTGCTTCAGGTCATCAAGAGCAATCACATATCGTAATT
GGGTTCGACAATTTGGAGGTTCCACCGAGATCGAAAGACTGGGTTTCCCCTTCGGCTTCC
TACATCTCTGCAGCTATCAGCACAAACATCGCGCACAGGTAAGCATTCTTACCATTGTTT
GTGCCTGATGTGTTGATGAGACGTGGTTGCCGAAGAACTCCCGGCCTCTTCGATTTAAGG
TGCAAAATTTGCGATTTGTTAGACCTAAATAATTTCAGGCGGCTTAGTACTGTAAGGTA
TAGACGTCTTGGGTATAAAGAATTGGCCAGAGATACTATAAGAAATAGTATATAGACGT
CTTGAGTATAAGGAATTGGCCAGAGATACTATAAGAAATAGTATATATACGTCTTAAGT
AAGATAAAGTCTTGCATGCATAAGGAATCGGCCGTTGAAAATAGAAATAGATAATTGAT
TGTTCTTATTTTTTGATTGTGTTGGGGCAGCCACTCACTTTGTTCTTGAATTACCATGGG

ACAGTCATATAGTTCGGCCCAGAAAGCCGATAAGGGGACTCCAGTTGCCTATATGGAAGC
CCATTTTGGCCAACACAGTGTTCGCTATCTGCGAAGCTGGACTAAATTAACTAAGGGCCA
GCCTTGCTCCTGGCCTCCAGACGGGTCCTTTAATAAAGATCGTATTTCCGCTCTTCAGGC
AGCAGTACATGCCAAATCTGAGCAGACTGGATGTGGTAGCTGCGTCTGCAGGAAGCATA
ACCTATCCCCACAAATAACGGCGCTTAATTTATGGGTCTCCGAAAGTCAGAAGCGACAAG
CGCAGAGTATTCTGGCCAGTCGACAGGACTCAGCGGCAAAGCTAATGGTGGCTCGGAAA
AAGAAAACGGAAAGGGAAAGAGATGCAGCTGAAGGAGTTGCCCTCTTAACAGGATGGAA
TGAGGACGAGGAACTGACGATGGCAGCAGCTGTCTCAGCGGTGGGGGTATGCCAATATCC
CAATATCGGCTCTGCCTCCACGCTGCCTTGTGTTTCCTTCTGTCCCCCAAATTCACCAACC
CCTTCAAGTGATTCCCATAAATTAACTGCAAGTAGTTCATCCGAACTCCCAGCTTCCTCG
GTGTCTGATGCAAAGGGAGTGCCCTCCACAACTACCCTTATGTCTGAATTGTCTTCGTCC
TCCAAGGGAGGAACACCTATGTTTGCTCCTGCTGACATAACCCTGTTAACAGGCACTCCT
GATCTCCGACCCTCTGCTCCGCCACCATATTCCAATTCCATGCCTACTGGCAGCTCTTGGG
TTTCGGAACCAATAATGAGTGACAATGGGGCAAACACGTTCCCCACGCACGAGTTTCCAA
CCCCTATGGGCGGCACATTTGACATTTATCGCCCATGGACGGCGACAGAATTAAAGGATT
TGATCTTACAAACCCCTACCCCAGACGAGGATTTTGCAAAGGCTGAAAGGTCATTAACGA
TTATACGCATGTGTCACAATCCTACTTTTATAGATTGGACTGTATTCTTGGAGGCGTGGT
TACCACCCTCCAAATCCTCCCAATTATTATCAGAATTACGCCAAAGGTGCGCCAGTACTG
ACAACACTAGACCAGCTCATCCCCCCGTGTTCGCAGTCTTCTCTTTGACCATGCCTTCAGC
TCCCCTCTCGACTCTCTTCCCCCTCCTCGCTCCCTCCCTTCCCTCCCCTCACCCTGACCATT
TCCCCCCTCCTTGCTCCCTCCCTTCCCTCACCCTGACCATTTCCCCCCTGCTCGGGTCATG
ACCCAACTGTACAGCGCCTTGGGACGTCCTCCTGACGTGAAAGGCGCTTTATAAATGGAA
TCTTGTTGTTGTTGTTGTTGTTGTTGTCATTATACAAATTTTACAACGTCTGTATCCCCA
GATCTCTGATTGGACTAAAATCAGCAGTTGCACCCAGAAGAAAGGGGAAACTCCTGACG
AATATGTTGAGAGGTTTTCGAAAATCTTTTCACGATATTCAGGGATTCAGAATGTAGCC
GATAACCAATCGCCCTTCGTGGCAGCGCTGGTGGCTGGTCTTGAAAAAGACTTGCAAGAA
ATGTTTAGACCCTTATGTATTGGTTGGGAAGGCAAGGGAATGACAGACATACTACCGGT
GTTGAGACACTGTCACAAACAGCTTGAAGTAAAGAAGGAAAAAGGGACCAAAATGGCCT
TTGAGGTGGCGGTGTCTACGATTGCTGATGGGTTGACGTCAGCTGCGCGGGGATCGAGCC
CTGCGTTAGCCACAGGTACACATGCACCTGACACCATGAGTGGGCTTATGCCTACCACCC
CTGCCACCGGTCCCACCATAATGCAAAATGTCGCACCTCCCGGAGCATGGTTTTGCCCGG
GAGGCATGATACCTCAAATGAGAGGCTTCCAAAAGCGGAGACCACGAGGGCAGGGTAGG
GGAAGAGGAGGATGGGGCAGAGGGAGAAATAATTTTGGGTACAACCATGGGTATATCCG
ACCTGCCTTTCAGCAAGCCTGCCCTCCCCTTCCTCGGCCTCAGCAAGTTCCCGGGGACCCT
AGTCAGAATTGCTTTTCGTGCGGGGCATATGGACATTGGTCCCAAGATTGCCCCACTAGG
AGTCAGAGATCCCAGGTCCAGTTTTACCCCCAAGATACGGCTGCCTACTCGCCCCAGGGA
TATCAGCAGGCACAAACACGGTTCCAGCACCCAGCACCGACGTATCAAATGCCTATGATA
ACTGAAGGAATGCCACCACAATGACTCTCAATGGCCCCAATGACTCCCCAATCCCATGTT
ACCTCCAAATCCTTGGAAATTCCCGATCCCTGTATTCGTCTGACCGTAGACTGGGAGGAA
AAGACGTTGCTTGTCGATACAGGGGCCAGCTATTCAGCCTTAAAAGATTCAAATTCCATA
CAACCAGGCAATACTGTAGCAGTAGTGGGAATTTCTGGGAAATCTGTGCAGATGGCCAA
GACTCAACTGGTTGAGGTAAAAGGGGATATAGGTGACAGTACAATTGAGGGTTACCACC
AGTTCTTGGTGAATAATGAATTACCAATTGACCTGTGTGGGCGAGATTTGTTATGTAAA
ATGGGAATAATCATCCAATGTACCCCGGATGGCATTACTTTGTTGGCACCTGAACGTGAG
TACTCAAAACTATGCTCCTTAATTTCTCCCAGCCCCCAGAAACTGCAATCCCCAGGTTAT
TTTTGCTGGACATTGCTAAAACCTGAGGTGTTATCCTCTGTCATGTCCCTTTATGGTGAC

GAACCACTCCTTGAATGTTATTTTTAGAGCATGGTTTGACAGCACCCACTTCCACATTGC
ATTGCACGGCTTTTTATGTTGAGAATATGGAGGTGGACATAACTCGGTTCAAGGAACAC
GTAGCGGTTGTTTCCACTATTCAGGCTCACTTTTTCTATCTGGGACCACAGGGTGTTGCA
GCTGCATGTACTCTGACAACGGAGGAGCAGCTTTATTTTAAAGGTAATAATTCCGACCCT
CATGTAACATTAAGAGTAGCAGACGGGTTTAGCCCAAGGGACTTAGGGCACATGGTCAG
GAAGGCAAACTGGTCGTCAGATTTCACTGAACTTTGCCCTCTAGTTTGCCCTGGCATTTT
ATACGACCCAGCTTTACGGATTTATAAGTTGTGTCTAGGTCCCCTCCCACTTTTTACTAT
GTTCGAGTTTATCCAACACACTCACACTATATCTTCCCATGTTCTGCCTCTTCGGCCGGC
AGATCCAGAGTGGTTGGAAGATATAGACTCCGTCCTATGGACAACACACCCCAATGATGT
AGGGAAAATACTGACGGCAGCACCCTTCAAGGTACAGGTGTACCCGGAAAAACCCCTTCC
CTACCGCGCGCAGTATCCATTAAAAAAGGAAGCAGAGGAGGGAATACGAGCAGTAATAG
AGAGCTTGTTGAATCAGGAAATCATTATTAAATACCGTAGCCCCTGTAACACTCCCATTC
TACCAGTCCTTAAAGCAGATGGAAAGAGATGGCGCTTTGTCCAGGATTTAAGAGCGGTT
AATGATGTAGTAATACAATGTTGTCCTGTAGTTCCTAATCCGGCGACTATTCTGACAGCC
ATTCCCGCTGTTTCGGTTTGTTATTCAGTTGTTGATTTATGTTCCGCATTCTTTTCAATC
CCAATTCACCCTGAGTCCTACTACTTGTTTGCTTTTACATACAAGGGTGAACAATATACG
TGGACCAGGTTACCCCAAGGATACTGTGAGAGTCCCACTATCTTTTCTCAGACTTTGCGG
CATGACTTGCAGGATTTGCGCTTTCCAGGGAAAAGTACCCTAATCCAGTATGTAGATGAT
TTATTAATTGCGAGCCCATCCGTGGAGGATTGTGAGAAAGATACCAAATACCTCTTGCA
GCAATTGGCGGTAAAAGGCCATAAAGTGGCGAGAGATAAATTACAGTTTGTAAGGCCAC
AGGTCCGTTATTTAGGACACGATTTGTGTCAAGACACTCGTCAACTTTCCCTTGATAGGA
TAGAGAGTATTAGGAAAATTCCCAAACCATCCACAATCAGGCAAATGAAACACTTTTTA
GGGATGGCCAATTATTGCAGGCAGTGGATTAGCAATTACGCTCAACTGTCTAAACCCCTG
ACTGAAATGACCAAACCGACCTCCAGCCTAGAACCCTTAGTGTGGAAGGCGGACCAAACC
TTTGATCAGCTCAAAACGGCTTTACAGAGCGCCCCCGCTTTAGGCATTCCCAATTATGAC
AAGATATTTAACTTATTTGTGCATGAAATCAAAGGATTCGCGCAAGCAGTCCTCACTCA
GGAACATGGGGGAGGCCAGCGCCCGGTTTCATACTTCACTTCCTAGTTGGATCCGGTAGC
CAGTGTTCTTCCAGGATGCCTGAAGGCTGTTGCTGCCACATACTATGCAGTTATGCAGGC
TGAAGGGTTGACGTTAAATCACCCCCTTATCGTGCATGTCCCGCACTCGGTCGAGGCATG
TCTATTTCGACATGCTACTCAACATTTAACAAGTGCCAGGAGCAGCAAGTATGAGGTGG
GTCTACTATCCAAACCAAATTTAACTATTAAACGTTGCACTGTTCTAAACCCAGCAACCC
TTGTGCCCATAGAAGAGGAAGGGGAACCCCATGATTGCCTTAAAGTCATTATATTAGAG
ACAAAACCTCGCTTAGATTTGAAGGAGAATCCCTTGCAAAATGCAGACTTTGAATGGTT
TGTAGATGGATCAGCTTGTAGGAACGAGTTCGGTCAACCCTGTGCAGGGTATGCTATTG
TATCAGCTACCACTATTATTGAAAGCGACTCGTTACCCTCCACCACATCTGCGCAGGCTG
CAGAGCTAACTGCTCTGAAGCGAGCATGTGAATTGGCTGAAGGGCAAACGCTCAATGTG
TTCACTGACTCCCGCTATGCATTCGGAGTATGTCATGATTTCGGTCAATTGTGGAAAAAC
CGGGGGTTTCTTACTTCAAGTGTGTCCCCCATTAAACACAAAGACCTTATAGTAGGTCTT
CTCACCGCATTACTACGGCCAAAAGCCATTGCGGTCATAAAATGCGCGGCCCATACTAAC
CAAACTGACCCAGTCTCTAAGGGAAATGCTCACGCAGATGCTGCTGCTAAAAATGCTGCT
TTAAGTAAGCCTGTAAAGTTACTAACATTACAACCGACTATCAAGCACCCCGCTGTCACT
GATACAATGCGTGGTGAGATATTTCGGATCCAATATGAGGCCCCTCTGGAGGAGAAGCA
ACAGTGGACCCGCCTACATTGTTTCATTAACGACCTAGGGATTTGGGTCTCGAATGAATT
AAAGGTAGTCCTGCCCCGACAAATGTTCCCATGGATGGCACATGTGTTGCATAATTTTAC
CCATGAAGGGACCCAGTCCATGTATAATTGGATAACGAAGTCGTGGTTTGCCCCAGGATT
AACCCCTTTTCTCCAGCAGGTTGTGAAATGTTGTGTAATATGTCAAAGGTTTAACCCAGG

CAAGGTTGACGATGTGACTCCGGCGTCCCATCCTCCCCCTCAGGGTCCCTTTGCGCACCTT
CAAATGGATTTTATTCAGTTACCAAAAGCCGAAGGATATCAGTATGTGTTGGTTATTGT
GGATATGTTTTCTAAATGGATTGAAGCTTTTCCTTGTAGGAGGAATGATGCCACGACAA
CGGCCAAAATATTGTTAAGAGACATCCTTTCTCGGTTCGGCATACCATCTCGCCTTTCCT
CCGATAATGGTCCTCACTTTATTGCCACGGTAATAAAGGACATTTGCAAAATGTTGCAA
ATAAAACAGCATCTGCATTGTGCATATCATCCCCAATCTGCAGGGGCTGTTGAAAGGGCT
AATGGGACGCTAAAGTTGAAATTGTCCAAACTGTCTGAACAGACAAAATTAAATTGGGT
AACAGTCTTGCCATTAGCCTTGATGCAAATGAGGAACCATACGAATGCAAAAAGCAAGC
TGTCTCCTCACGAGATCATTTTCGGAAGGAGCATGCCCATGGTTTACTCCGCACACATTG
AACCGTTGCCTGACGTGCATGCCCTCGAGGAAGGGATGATGCAATACTGCATTGCGTTGA
CTAACACCCTATTTAGTTTTAATTCACAGGTTCGAGTGCTCCAACATCCACCGGCATCTG
ATCAATGCCACAAGTTTCAGCCAGGCGATTCTGTTTACATCAAGACCTTCAAACGAAAGA
ACGGACTGAGCCCGCGCTGGGAAGGACCGTTCCAAGTTCTATTGGTGACCCACACTGCTG
TGAAGGTTCGAGAAAGGTCTACGTGGATTCATGCTAGTCACTGCAAGCAAGCCCCAGCGG
ACTCCGGCGATTGCGAAAAAGCCTAACGTTGTTACTTTTTCATTTTTTTTGACGGACTTT
GCTTCATTATGGGGTTGTTGTTTTGTATGTTTTTATTGTTTTTGCTATGTTGCCATACTT
ACTCTCTTGTCCCAGGGGGAGGTGTTTACTGATTTTTGATCTCCTTAAACCGTGATCTCA
CCGTATCTGATGATCAGTCGGAAGCCTGTTCTTCCGCCATTTGTTACACTGATATACCCC
TTGAGAAACGATCCTACTTGTTTAATGTTCATTTTTTGTTGTGGAATGCTCAGGATAGA
TCATACGCAGACGATTTGAGCTGTCCTAAGGATTCCGGTCAATGTCAATCCGTCCATAAC
AACTCCATGATTATGTGTTATTGTTCCATTTATTATCACTGTTCTTTTACCGAGTCTTGC
TTGTACTCTACTGAACGCGCCCGGTTTTCCAACGAAACCCAGCTCGTGTTTTTGCGTCCT
GGTACCTATAGGGCTAGAATTTATACAGGGGTGTCTCAGGACTGGCACGAGGGTTTAGT
TGAGCCTCACTTGCCTTCCTCGTCACTGGTCTCACTTCCTCGTGACGTTCCATTTGTTGAC
ATGTATCCCTTTAAAACTGCGCACCAACATTTGTTTGGTGACTTTTTACCAGCAGATTCT
CCCCAGCCTTTCCAATTGCGGAATGTTTATATTCAAATGCTCCAGACTGTGAGCTTGCAC
AGAAATAAATCCCAGCATCATGTATGTGGATTGTCCCCACCGTCAGTCCAGATGGGGTAC
CCTATGATGGCTTTCCCATTGTCTACCTGTGAGCTTTGTTCCCTTTTTCATTTGCAAGTT
AGGCAAACCTCACCTTGTTCCCTGTTACACCAAGAATTACACGAATTGCAGTGTGCTCGT
CAATCCTGGAAGCCACGAATGTGTAATCGTGACTGCGGCAAGCCCCCTAAGATTGGCACT
GCCACTATGGTCTATGCGGGTTTTTCGTTTTGCGTCGAGTCCTTTTCCGGGACTGAACCA
CTGGGGTTTCTGGATTCCCACCTTTGCAACACCACATATCCTTATGCTGCGGTGGCTGTT
TTCCCTTCTAATTTCGGGGATTGCCAGGTCACTTTATCATCACATATTCACAGGATGATA
ACATACTCCGTTAATCAGCGCTGTGCCATTTCCCCGATTCATAAGGATTTTTATTGGGTT
TCCAATTCTACTGTCTATGCTTACTTACCAGCAAATTTCAAAGGGAGATGCGCTCTCTTT
CATTATTTTCCTATGATTCGTCTAACCAATACCCTGCCTTCTGCCCGGGCCCCACTAGTCC
TAAATACCTCCAGCCTACTCCCTCCCCGCCAGGTTCATCACTCTCGCAGAGCCGACCCTTT
TCACACCCATTCCCAGATCCTGTGGAATCGCATGCTCGGGGCTCTTCTCCCTAATTATGG
AGTAATGTGCGCCCTTGACCAGATTGCCATTCTCTCTCAGGCGGTCTCCACATTGGCCTC
TGATACATCTGCGGCATTGGCTCTCGTTAACAAAGAGATGTCTGAGATTCGTCTATAGGC
TATGCAGAACCGTATGGCCCTCGATTATGTTTTAGCTGTGACGGGAGGAGTGTGTAAGG
TCATTGGACCTGAATGCTGCATAACAATTGATGATTTTTCTGGTTCTATCTCAAACATTA
CCAAAGAGATCAATCAAACAGGGCATGATGCGAGTAATATCATCATACATTCTGGAGGA
GGATTACTGGATTGGTTGGGTAATTGGTTGCAAGGTATGGGGCTAGGATGGTTGGGCAG
TGCTGTCCAGGTTCTATTGGAAATAGGCCTAATCCTTGTTTTTATTGTTCTCATCACTAA
ATGTGCCCTCATTTGTTGCACACAATGTTGCAAAAAGACTACACGCACCATGATGACCAT

GAACATGGTCCACGTTAACCAACACCTTAGCCCATTTTCCCCACGTGATAAATGTGACCT
CGAGATGCCCTATGCCTTTCCGGATTCCCTCTCGGAAAGCAGTACACTCGAGGGCTAGAA
AGCACGGAAGGGTTATCCCAGGGATAAAAGGGAGGAGTGTGGAAGGTGAATTCCGCACA
TTCTGCAGAGTTAGCAAAATGAACAGGACGTGAGGACCTAAGACCAAAAGGGCAAAAGG
CCAGGGGGGCCCCTGGCCTTAAGTGAAATATATATGTTGTTATGAAGTTGTCACGAATGC
TCGTGATTGGCATTGTTTAAAGATAGATAAGCAAATGTCTAGTATCGGTACCGCTCCTG
AAGTCATGTCTGGAATCAGTTCTGGGGTCAGATAACATTCAGCAGCATCAGCATTTGTCA
TTCTTTAAAATGTCCTTTTCCTATATGTACTTAATTGTAAGGACAGAGTAATTGTCTTTG
TAAAACAGACATCCGGTCACAAGGAAGACTATGTCGAAATGTAACCAATTTAGAGGGTC
CCCTCATGATTATTGATTGACTCATAGTTCGATAAGATTTTGTATAAAGGTGAGGTGCT
TGCTCCCATTTAGTGTGTAGTGTTGGGAGCAGCACACGAGCAGTTGTTGCCACGATACTA
CACCCCACATGTGTGAAAATAAAACTTGTTAATTGAACGCCGCACTGACTCCGTTGTTGT
GCTTCAGGTCATCAAGAGCAATCGCATATCGTAATTGGGTTCGACA

>CmiERV_scaffold 2324
GAGGAGAGAGAGGAGACTGAACGTATATTAGCGTTAATACAGTCTCCTGGGAGTGCTTG
GTTGACATTAAGAGTGAATGGAATTGACATACCGTTTTTATGCGATTCAGGAGCATGTA
GAACGGTTATTACGATGAGAGACGCAACTTTTCCTTATAAAAAGAGTCTGGAAAAAATT
GATATTCGATCAGCGTCCGGAGACTTAGAAACACAATGCGTCACGGAACCTCTTGAATTC
TGTTACAAGAATAAAGAGACTGTAGAAGCACAAGTGGTTATATCAATGACATGCCCGGT
TAATCTACTAGGGAGAGATGTGTTAACTAAACTCAAGTTAGGAGTGATACCTATGGGTG
ACGACCGATGGGAAGCAGTTAAGGTTACGTAAAAAAAAAGATTGAAGAAACCCTAATTA
TCCAAGAAAGGGGGGAACCACAATACTATTATGCCCTACAGTTAAAGGAAGGGCCAGGA
CAATTCCAACAGGAACTGCTTAGATTGGCACACCAGCGGGTCATGGAACAAAGTGACTTC
CACCTGCCTGAGGATCTGCACTGTACCATGTGGTACCGTGAAAAACGGGGAAGTTCGTTG
CCATATGAGAGACATTTCAAATTGGGAAGTTCCACACGATTAACGATTCAATGTATATA
TTGGAATGCAACTGATATGGGAGGGAAAGTTTTACTCCAACCGTTAGATGATCAGCTAT
ACCAGGGAGAAGGGAACCCCCATGTATCAATTGCAAAGAGATGCAAAAAGTCATGGCAA
CAAGTGGGTAACCTGGTAGATTGTGGGATAAAAGCACAGGATTGGGAAGAGACTCAGGA
TGCAGGAATATGGTATTCCCCGAGGACAGGTCTCTACTCACAATGTTTGAATTGGGTGTA
TCAATGTGAAAGAGTTAGGAAGATGCACTCAGGGAAGATGGTTCCCTGACTGTTAGGAG
TGGAAATAGAACAGGAGGCGGAGTTGAATGCTCTGCCTGATGCTCTATGGTCCAAAGGA
TCAGCTGACGTAGGACTAATAAAAGGGATTGAACCTATTAAAATCATACCGAAAGGAAC
ACACCGCCCGTACCAACGGCAGTATCCTATGAAACCAGAGGCCATTGAAGGAATTGAACC
CATAATTATGGAACTTAAGGAGAAGGGCATCATAGTGGATTGCCCTGACTCTCCATGTA
ACAGCCCCCTGGTCCCAGTAAAAAAGGTAGCGCCATCACAAGGGTGGCGATTGGTTCAGG
ATTTACAGGCTGTAAATAAGGCAGTGATTCCCCGGACCCCAGTTGTACCCGACCCCAATA
CCTTGCTGAACAATTTGCGACCTGATACAGCTTGGTTTACAGTAATTGATTGAGCAAATG
CGTTTTTTTCTATTCCGGTTGACAAGGCCTCCCAATTTTGGTTTGCCTTCACATTTTTGG
GAAGGAAATATATGTACACCAGATGTCCACAGGGATTTGTTGAATCCCCGGCCATATTCT
CACAATGTATGGAAAGTAATCTGGCAAAATTTGAGCCCCCGGGTGGCAGTCAAGTTTTG
TTGTATGTAGATGACATACTCATATGTTCCCCGTCTAACGAAATTTGCCGCCAAGATTCT
CTGGCTTGCTTAAGATTCTTGGAAGAATGGGGCCATAAAGTAAGCCAAAATAAACTACA
ATATTGTCTACCCGAGGTCAAATATCTGGGCCATACAATTTCTAGGGAAGGGAAATCCTT
ATTGAAAGACAGAAAGCTTGCGATAATGCAAGCTCCGCGGCCCGAAACTAAACAACAGA
TGATGTCATTTTTGTGGATGACTAATTTCTGCCGCGCTTGGATCTCTTCATATGCGGAGC

TGACTCAGCCCTTGTTAGGGTTGATTTATGATACCCCTATGAAGGCACAGGATGCAATTG
AGTGGAACCCAGAGGCTATAAGGGCATTTGAAATTATAAAAACGATTTTATCCAGGGCA
GAAACTTTGGCACATCCTGACCCTGTAAAGCCATACACTCAAACTGTCTGCGTTAAAAAT
GAGTTTATGGTATCTGTCCTCTTACAACCATTTGGAACGCGAATGCGACCCGTGGCTTAT
TACTCACACTGACTGGATCCAGTGGCTCGAGGCCTTCCTTGCTGCTTACAAGCAGTAGAA
GCAGCAGCTCTGGCTGTTCGCGCATCATTGCCCCTGGTCCTGTACCAACCATTGAAGTTG
TTGGTTCCACATGCAGTTTCGGTATTATTGCTTCAAACTAAACATGCCTTCCTATCGCCA
GCACGCCAGCTATCCTGTATGGCCACATTGCTGACCTGCCCAAATTTAACCATTGAACGA
TGCACGGTGTTGAATCCGGCAACCTTGCTGCCAGTACCTTCCGAGGGTAGCCCTCACGAC
TGTTTGGAAAAGACCTCACAGTCAGTAGCGGTGCGAGTAGATTTGGCGGACACAAGGTT
TGAGAATGCAGATGTGACATACTTTGTGGACGGTTCATGCAAAAAGGACATGGTGGGCC
GTTCCCAAAGTATGTATGCTGTATGCACTCAGGACACGGTAGTAGAAGCAGCCAGACTCC
CAACAGGATATTCGGCACAAATGGCTGAACTTGTGGCGCTTACTCGAGCTTGTGTACTGG
GGAAGAGAAAAATTAATAACATCTATACAGACTCACAATATGCCCATTCTAATTGCCAC
ATATTTGCCGGACAGTGGCGAAACCGGGGTATGGTTACCTCCACAGGTAAACCAATTTCC
CATGCTGATTTAATATTGAACCTAATTGATGCATTTCAACTCCCAAAACAATTAGCAAT
ACTTAAGTGTGTTGCCCACACCTCTGCGAAAGACCTGGTGTCAAGGGGAAATGCCTTTGC
AGATAAAACTGCCCAGGCAGTGCCCCTCGCAGAAGTATTAGTGTCACAAACACCACCCTC
GATCGACATGACATTCATTAAAACGGTGCAATCAGAGGCAGGCACCCTTGAGAAGCGAG
GATGGAAAAACATGGGATGCAAACTAAACACATTCGGCATATGGGTTGGAAACACTGGA
CTGCCAGTCTTACCACGATCATTGTTTCCCATTGCAGCAGGATTGAGCCACGGCAAATGC
CATGTCTCAAAAGGAGGGATGAGGACAACGATAGACCAACAATATACAACATTCGGACT
AACCACTTATTTACCAAAATTTTGTCAACAATGTATAACATGTTTACGCAATAACCCACA
AGGACAACTAAAATGTAAACAAGGGAAGATTCTATTGGCAAAGGAACCCTTCTCTCATT
GGATGATGGATTTCATCCATCTAAACGTATGCGAAGGAAAAAAATATTGCCTAGTAATG
GTATGTCCTTTCTCGGGTTGGGTAGAAGCTTTCCCCACAACCCATGTGGATGCAGTAAGA
GTAGCAAAGATATTATGCAGAGAAATAATACCAAGATGGGGTATCCCGCGAATATTATA
TAGTGATAATGGGCCGGAATTTAAGAATGAGGTCCTGAGAAATGTGGGGATTCACTTGG
GGATAACCTTAAAGAACAACTGTGCCTACCATCCAGAGTCTGCAGGAATGGTAGAAAGA
CATAATGGGACACTTAAAAATAAGTTGAAGAAAGCCATGGAAACCACAGGAAAGGGTTG
GGTGGCATGTCTACCATTAGCACTGACAAGTATGAGAATTACACCTGGGAGCAAGAAAC
TCCCCGTATGAAATTGTTATGGGTAGAGCATATTACATGCTTGGAGAACGTACAGAATT
GGAGGAGGCCGGACTTGCAGGATATATGAAAAAAATGTTTCAGAACAAAATGCCTATGA
GAAACGTATTACCTCCCCAGGAACTGAATGAGAACACCGAGCCATGCCCACTGCAGCCAG
GAGATTGGACACTGATTAAAGACATCAAAAGGAAGCATTGGCATCAACCAAAGTGGACA
GGACCATATCAGGTGCTACTAACCACACCAACAGCAGTACGGATTGCAGAACGAAACACC
TGGGTCCACGTTAGCCACTGC
