# Supplementary material for: Extensive retroviral diversity in shark
Source: Retrovirology. 2015 Apr 28;12:34. doi: 10.1186/s12977-015-0158-4 (PMC4422223; doi:10.1186/s12977-015-0158-4)
Supplement: Additional file 2: Table S1. — The representative retrovirus sequences used for phylogenetic reconstruction. [file 12977_2015_158_MOESM2_ESM.pdf]

Table S1. The representative retrovirus sequences used for phylogenetic reconstruction

Name	Accession No./Source
Bovine foamy virus (BFV)	NC 001831.1
Equine foamy virus (EFV)	NC_002201.1
Feline foamy virus (FFV)	NC_001871.1
SFVspm	EU010385.1
SFVgor	HM245790.1
SFVcpz	NC_001364.1
SFVmac	NC_010819.1
SFVagm	NC_010820.1
Bovine leukemia virus (BLV)	NP_056895.1
Human T-cell leukemia virus type 1 (HTLV-1)	NP_057860.1
Human T-cell leukemia virus type 2 (HTLV-2)	NP_041003.2
Bovine immunodeficiency virus (BIV)	NP_040563.1
Human immunodeficiency virus type 2 (HIV-2)	NP_663784.1
Human immunodeficiency virus type 1 (HIV-1)	ABK51636.1
SIVcol	AAK01033.1
SIVcpz	ABU53017.1
Jembrana disease virus (JDV)	AAA64389.1
Caprine arthritis-encephalitis virus (CAEV)	NP_040939.1
Maedi-visna virus (MVV)	YP_536867.1
Equine infectious anemia virus (EIAV)	NP_056902.1
Feline immunodeficiency virus (FIV)	NP_040973.1
Lymphoproliferative disease virus (LDV)	AAA62195.1
Avian leukosis virus (ALV)	YP_004222728.1
Murine endogenous retrovirus type L (MERV-L)	CAA73251.1
Python molurus endogenous retrovirus (Python-molurus)	AAN77283.1
Mouse mammary tumor virus (MMTV)	NP_056880.1
Jaagsiekte sheep retrovirus (JSRV)	NP_041186.1
Simian retrovirus 2 (SRV-2)	AAA47562.1
Simian retrovirus 1 (SRV-1)	AAA47732.1
Snakehead retrovirus (SnRV)	NP_043924.1
Walleye epidermal hyperplasia virus type 1 (WEHV-1)	AF133051
Walleye epidermal hyperplasia virus type 2 (WEHV-2)	AF133052
Walleye dermal sarcoma virus (WDSV)	NP_045937.1
Feline leukemia virus (FLV)	NP_955577.1
Moloney murine leukemia virus (MMLV)	NP_057933.2
Baboon endogenous retrovirus (ERV-Baboon)	BAA89659.1
Baboon endogenous retrovirus (ERV-Baboon 2)	AHZZ01047987
Gibbon ape leukemia virus (GALV)	NP_056790.1
RD114 retrovirus	NC_009889
Rhinolophus ferrumequinum retrovirus (RfRV)	JQ303225
Tursiops truncatus endogenous retrovirus (TTERV)	ABRN02237111
Koala retrovirus (KoRV)	AF151794.2

Porcine endogenous retrovirus C (PERV-A)	EU789636
Porcine endogenous retrovirus C (PERV-C)	HM159246
Xenopus tropicalis endogenous retrovirus 2 (XTERV2)	HM765512
Zebrafish endogenous retrovirus (ZFERV)	AF503912
Atlantic salmon swim bladder sarcoma virus (SSSV)	DQ174103
Rabbit endogenous lentivirus type K (RELIK)	Ref. 1
Gray mouse lemur prosimian immunodeficiency virus (pSIVgml)	Ref. 2
Sloth endogenous foamy virus (SloEFV)	Ref. 3
Mustelidae endogenous lentivirus (MELV)	Ref. 4
HERV-S	Ref. 5
HERV-L	Ref. 5
HERV-FRD	Ref. 5
HERV-Hconsensus	Ref. 5
HERVH-RGH2	Ref. 5
HERV-Fc1	Ref. 5
HERV-W	Ref. 5
HERV-E	Ref. 5
ERV-3	Ref. 5
HERV-T	Ref. 5
Xen1	Ref. 5
HML1	Ref. 5
HML2	Ref. 5
HML3	Ref. 5
HML4	Ref. 5
HML5	Ref. 5
HML6	Ref. 5
HML7	Ref. 5
HML8	Ref. 5
HML9	Ref. 5
Gallus gallus endogenous retrovirus LA (GGERV-LA)	Ref. 6
Gallus gallus endogenous retrovirus 10 (GGERV-10)	Ref. 6
Zonotrichia albicollis endogenous retrovirus type A (ZAERV-A)	Ref. 6
Coelacanth endogenous foamy virus (CoEFV)	Ref. 7
Galeopterus variegatus endogenous lentivirus (GvaELV)	Ref. 8

References

1. Katzourakis A, Tristem M, Pybus OG, Gifford RJ (2007) Discovery and analysis of the first endogenous lentivirus. Proc Natl Acad Sci USA 104: 6261-6265.
2. Gifford RJ, Katzourakis A, Tristem M, Pybus OG, Winters M, Shafer RW. (2008) A transitional endogenous lentivirus from the genome of a basal primate and implications for lentivirus evolution. Proc Natl Acad Sci USA 105: 20362-20367.
3. Katzourakis A, Gifford RJ, Tristem M, Gilbert MT, Pybus OG (2009) Macroevolution of complex retroviruses. Science 325: 1512.
4. Han GZ, Worobey M. (2012). Endogenous lentiviral elements in the weasel family (Mustelidae). Mol Biol Evol (in press).
5. Jern P, Sperber GO, Blomberg J. (2005). Use of endogenous retroviral sequences (ERVs) and structural markers for retroviral phylogenetic inference and taxonomy. Retrovirology 2: 50.

6. http://bioinformatics.cvr.ac.uk/paleovirology/site/html/retroviruses.html
7. Han GZ, Worobey M. (2012). An endogenous foamy-like viral element in the coelacanth genome. PLOS Pathog 8: e1002790.
8. Han GZ, Worobey M. (2015). A primitive endogenous lentivirus in a colugo: insights into the early evolution of lentiviruses. Mol Biol Evol 32: 211-215.
